# Characterization of Novel *WFS1* Variants in Three Diabetes Pedigrees

**DOI:** 10.1111/1753-0407.70114

**Published:** 2025-07-10

**Authors:** ChangQing Liu, HangYu Fang, Dong Wang, YiPing Cheng, Ping Shi, ChunXiao Yu, XiaoHong Li, Hui Zhao, Wei Hou, ZhenKui Guo, Chao Xu, QingBo Guan

**Affiliations:** ^1^ Shandong First Medical University and Shandong Academy of Medical Sciences Jinan, China. Endocrine and Metabolic Diseases Hospital of Shandong First Medical University. Shandong Institute of Endocrine & Metabolic Disease Jinan Shandong China; ^2^ Key Laboratory of Endocrine Glucose & Lipids Metabolism and Brain Aging, Ministry of Education; Department of Endocrinology Shandong Provincial Hospital Affiliated to Shandong First Medical University Jinan Shandong China; ^3^ Shandong Key Laboratory of Endocrinology and Lipid Metabolism Jinan Shandong China; ^4^ Shandong Institute of Endocrine and Metabolic Diseases Jinan Shandong China; ^5^ “Chuangxin China” Innovation Base of Stem Cell and Gene Therapy for Endocrine Metabolic Diseases Jinan Shandong China; ^6^ Shandong Engineering Laboratory of Prevention and Control for Endocrine and Metabolic Diseases Jinan Shandong China; ^7^ Shandong Engineering Research Center of Stem Cell and Gene Therapy for Endocrine and Metabolic Diseases Jinan Shandong China; ^8^ The Cancer Prevention and Control Hospital of Tai'an Tai'an Shangdong China

**Keywords:** diabetes, gene mutation, MODY, *WFS1*, wolframin protein

## Abstract

**Background:**

Mutations in the *WFS1* gene are implicated in Wolfram syndrome (WS), Wolfram‐like syndrome (WFLS), and maturity‐onset diabetes of the young (MODY). Wolfram syndrome 1 (*WFS1*) is a diabetes‐related gene encoding wolframin, a glycoprotein with nine transmembrane domains localized in the endoplasmic reticulum. However, the relationship between *WFS1* mutations and their associated phenotypes remains incompletely understood, requiring additional patient data collection for further investigation. Here we collected and analyzed clinical data from three diabetes pedigrees, and to assess the genotype‐phenotype correlation.

**Methods:**

High‐throughput sequencing was employed to detect *WFS1* gene mutations, followed by pathogenicity and conservation analysis using bioinformatics software. A three‐dimensional wolframin protein structure was constructed to investigate the potential effects of the mutations. Moreover, the distribution of *WFS1* mutations and their associated clinical phenotypes were analyzed by summarizing genetic variations of the *WFS1* gene recorded in the Human Gene Mutation Database.

**Results:**

Four heterozygous *WFS1* mutations were identified in three diabetes families. Among these, c.1523_1524del/p.Y508Cfs*34 was identified as a frameshift mutation, while the others were missense mutations. Bioinformatics predictions revealed that c.766A>G/p.K256E is a benign and novel mutation, whereas the remaining mutations were classified as pathogenic. Furthermore, c.985T>A/p.F329I was validated as a MODY‐associated mutation within a specific family. A comprehensive summary of all reported *WFS1* mutations indicated that mutations associated with WS phenotypes are approximately 18.7 times more frequent than those associated with MODY phenotypes. Missense mutations accounted for the highest proportion of *WFS1* mutations associated with different clinical phenotypes, with the majority located in exon 8.

**Conclusions:**

This study identified a novel *WFS1* mutation, c.766A>G/p.K256E, expanding the known mutation spectrum of the *WFS1* gene. The findings suggest that inactivating mutations and benign missense mutations are associated with more severe WS phenotypes compared to purely pathogenic missense mutations. Moreover, c.985T>A/p.F329I was validated as a MODY associated mutation. Finally, by summarizing the genotype–phenotype relationships of *WFS1*, it is concluded that the *WFS1* gene shows a different association with WS, WFSL and MODY.

## Graphical abstract

We first collected the patients with diabetes, including the clinical data and blood. Then the high‐throughput sequencing platform was used to detected gene mutation using blood samples. After screening the possible mutation sites, we collected blood from the patient's family members and performed Sanger sequencing for verification. And we analyzed their pathogenicity and conservation using bioinformatics software, and constructed a three‐dimensional Wolfram protein structure. Finally, we analyzed the distribution of *WFS1* mutations and associated clinical phenotypes by summarizing the genetic variations of the *WFS1* gene recorded in the Human Gene Mutation Database. We got results as follows: (1) c.1523_ 1524 del/p Y508Cfs*34 is a frameshift mutation, c.766A>G/p. K256E and c.985T>A/p. F329I are missense mutations. (2) c.766A>G/p. K256E is a benign and novel mutation, while the rest are pathogenic mutations. (3) c.985T>A/p. F329I can cause MODY in a family. (4) Phenotype and clinical presentations diverse in patients with *WFS1*‐associated disease. (5) The proportion of *WFS1* mutations corresponding to different clinical phenotypes were highest in missense mutations, mainly distributed in exon 8. We concluded that c.766A>G/p.K256E is a new WFS1 mutation that has never been reported before, and that inactivating mutations and benign missense mutations lead to more severe WS phenotypes than pure pathogenic missense mutations. We also verified that c.985T>A/p. F329I as a MODY‐related gene. Finally, by summarizing the genotype–phenotype relationship of WFS1, we concluded that the clinical manifestations of *WFS1* mutation‐related disease are diverse and require accurate differential diagnosis based on laboratory tests and genetic sequencing.

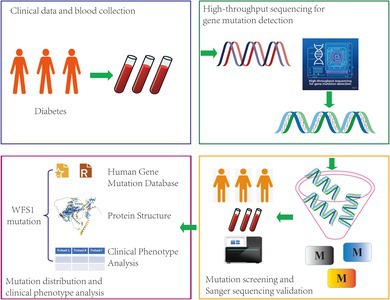

**Summary**

- We found four *WFS1* heterozygous mutations in three diabetes families, of which c.1523_ 1524 del/p Y508Cfs*34 is a frameshift mutation.
- We discovered a new *WFS1* mutation c.766A>G/p.K256E in a diabetes family.
- We verified that c.985T>A/p. F329I as a MODY related gene.

## 1 Introduction

A wide range of clinically rare diseases are associated with *WFS1* variants [1, 2]. This gene is located on chromosome 4p16.1 and comprises eight exons [3, 4]. It is highly expressed in brain tissue, pancreatic β cells, and the heart [3]. The *WFS1* gene encodes wolframin, a 9‐transmembrane glycoprotein of the endoplasmic reticulum (ER) [3, 5]. Wolframin is embedded in the ER membrane [6, 7] and plays a critical role in protein folding, translation, and the regulation of ER calcium homeostasis [7, 8, 9]. Wang et al. [10] discovered that insulin prohormone is transported from the endoplasmic reticulum to the Golgi apparatus by the wolframine protein as a receptor for vesicular cargo proteins. Mutations in *WFS1* lead to defects in the function of the wolframine receptor, preventing the transport of insulin prohormone from the endoplasmic reticulum to the Golgi. This leads to abnormal accumulation of insulin prohormone in the endoplasmic reticulum and induces uncontrolled ER stress [11], which impairs normal β‐cell function. In addition, the transport and processing blockade of insulin prohormone prevents its conversion into insulin, leading to reduced insulin secretion.

Wolfram syndrome (WS) is the syndrome most closely related to the *WFS1* mutation. WS is a rare autosomal recessive monogenic neurodegenerative disorder caused by mutations in the *WFS1* gene. The primary clinical features include juvenile‐onset diabetes mellitus, optic atrophy, diabetes insipidus, hearing loss, and neurological complications [3, 12, 13]. Approximately 98.2% of WS patients present with diabetes. WS‐associated diabetes is a non‐autoimmune, insulin‐dependent diabetes mellitus that typically manifests before 6 years of age [14] and is mainly treated with insulin. Optic nerve atrophy commonly occurs around the age of 11 [14, 15], while ~75% of WS patients develop diabetes insipidus by the age of 14 [13]. Symptoms of hearing impairment generally emerge during late adolescence [16, 17]. The disease has a severe prognosis with rapid clinical progression, with most patients dying from respiratory failure caused by brain stem atrophy in their 30s, and there is no effective treatment available [14, 18]. Active clinical follow‐up and supportive care can help delay the progression of WS [13, 19]. WS typically has an early age of onset and is a progressive disorder, follows a prolonged course, and is associated with a poor prognosis due to mutations in the *WFS1* gene. Wolfram‐like syndrome (WFLS) is a newly reported autosomal dominant disorder linked to mutations in the WFS1 gene. WFLS has similar phenotypes to WS, including optic nerve atrophy, hearing loss, and diabetes [1].

The *WFS1* gene has also been recognized as a Maturity‐Onset Diabetes of the Young (MODY) gene [20, 21]. MODY is a form of monogenic, non‐autoimmune diabetes caused by single‐gene mutations [20, 22]. Diagnosis of MODY must include clinical symptoms (early age of onset, family history, glycaemic profile) and genetic testing, according to the Standards of Care in Diabetes‐2024 [23]. Several genes have been implicated in MODY, including *HNF4A*, *GCK*, *HNF1A*, *PDX1*, *HNF1B*, *NEUROD1*, *KLF11*, *CEL*, *PAX4*, *INS*, *BLK*, *ABCC8*, *KCNJ11*, *APPL*, and *WFS1* [21, 22, 24]. Although *WFS1* mutations have been associated with MODY [25, 26], limited studies have explored the relationship between *WFS1* mutations and MODY.

To date, the Human Gene Mutation Database has recorded 604 mutations in the *WFS1* gene. For instance, Du et al. investigated 11 patients from seven WS families and identified eight homozygous mutations, including five novel mutations [27]. While previous studies have established that *WFS1* mutations are causative for diabetes [25, 28, 29], limited research has been conducted on *WFS1* mutations in China. The genotype–phenotype relationship of *WFS1* mutations remains poorly understood, and the pathogenic mechanisms are not yet fully elucidated.

In this study, a follow‐up investigation of three diabetes families with *WFS1* mutations was conducted. These mutations may indicate a role of *WFS1* in the pathogenic mechanism of diabetes, and alternative treatments should be investigated.

## 2 Subjects and Methods

### 2.1 Research Subjects

In Family 1, Proband A was an adolescent boy admitted to Shandong Provincial Hospital, affiliated with Shandong First Medical University, in July 2021 due to a 2‐week history of a short penis. The initial diagnoses included “delayed sexual development” and “complete Wolfram syndrome.” The parents of Proband A were not consanguineous.

In Family 2, Proband B was an adolescent with diabetes who was hospitalized at the same institution in 2022 due to a one‐year history of sticky urination. Proband B's parents were also non‐consanguineous.

In Family 3, Proband C was an adult pregnant woman with diabetes admitted to the Endocrine and Metabolic Disease Hospital affiliated with Shandong First Medical University due to hyperglycemia.

Detailed medical histories of the probands were recorded, including physical examinations and auxiliary tests. After obtaining informed consent from the probands and their families, peripheral blood samples were collected from Probands A, B, and C, as well as from Proband B's parents and the family members of Proband C for genetic testing. The study was approved by the Ethics Committee of Shandong Provincial Hospital, affiliated with Shandong First Medical University (Approval LCYJ: No. 2019‐147, July 2019). The research protocol adhered to the Declaration of Helsinki (2013, Brazilian revision).

### 2.2 Peripheral Blood Genomic DNA Extraction

Peripheral blood samples from Probands A, B, and C, as well as the parents of Proband B and family members of Proband C, were collected. Genomic DNA was extracted from these samples and processed for whole‐exome sequencing (WES) following fragmentation, splicing, amplification, and purification.

### 2.3 Whole Exome Sequencing and Sanger Verification

DNA libraries were prepared using a hybridization capture method. WES was performed using a high‐throughput sequencing platform to detect the exon regions and flanking intron regions (20 bp) of human exome genes. Sequencing data were aligned with the human genome reference sequence hg19 (GRCh37). Parameters such as coverage and sequencing quality in the target regions were collected. The average sequencing depths for Probands A, B, and C were 151.88×, 130.87×, and 162.99×, respectively. Regions with sequencing depths exceeding 10× accounted for 98.54%, 98.77%, and 98.48%, respectively; regions with depths exceeding 20× accounted for 98.33%, 98.48%, and 98.15%, respectively; and regions with depths exceeding 50× accounted for 96.46%, 95.11%, and 96.08%, respectively. Pathogenic or potentially pathogenic variants detected through WES were confirmed by Sanger sequencing, ensuring 100% coverage of the gene coding sequences. The pathogenicity of the identified variants was evaluated based on the guidelines of the American College of Medical Genetics and Genomics (ACMG).

### 2.4 Bioinformatics Analysis

The bioinformatics tools MutationTaster (http://www.mutationtaster.org/) and PolyPhen‐2 (http://genetics.bwh.harvard.edu/pph2/index.shtml) were used to predict the pathogenicity of the identified mutation sites [12, 13]. Conservation analysis of the mutation sites across species was performed using UGENE software [14]. Protein structure predictions were conducted using the Swiss Model (https://swissmodel.expasy.org/) and visualized with PyMOL.

### 2.5 Literature Search

*WFS1* mutations and associated literature were retrieved from the Human Gene Mutation Database (HGMD) (http://www.hgmd.org). The distribution of *WFS1* mutations across various exons was analyzed using the National Center for Biotechnology Information (NCBI) database (https://www.ncbi.nlm.nih.gov/gene).

## 3 Results

### 3.1 History and Clinical Features of the Proband

**FIGURE 1:**
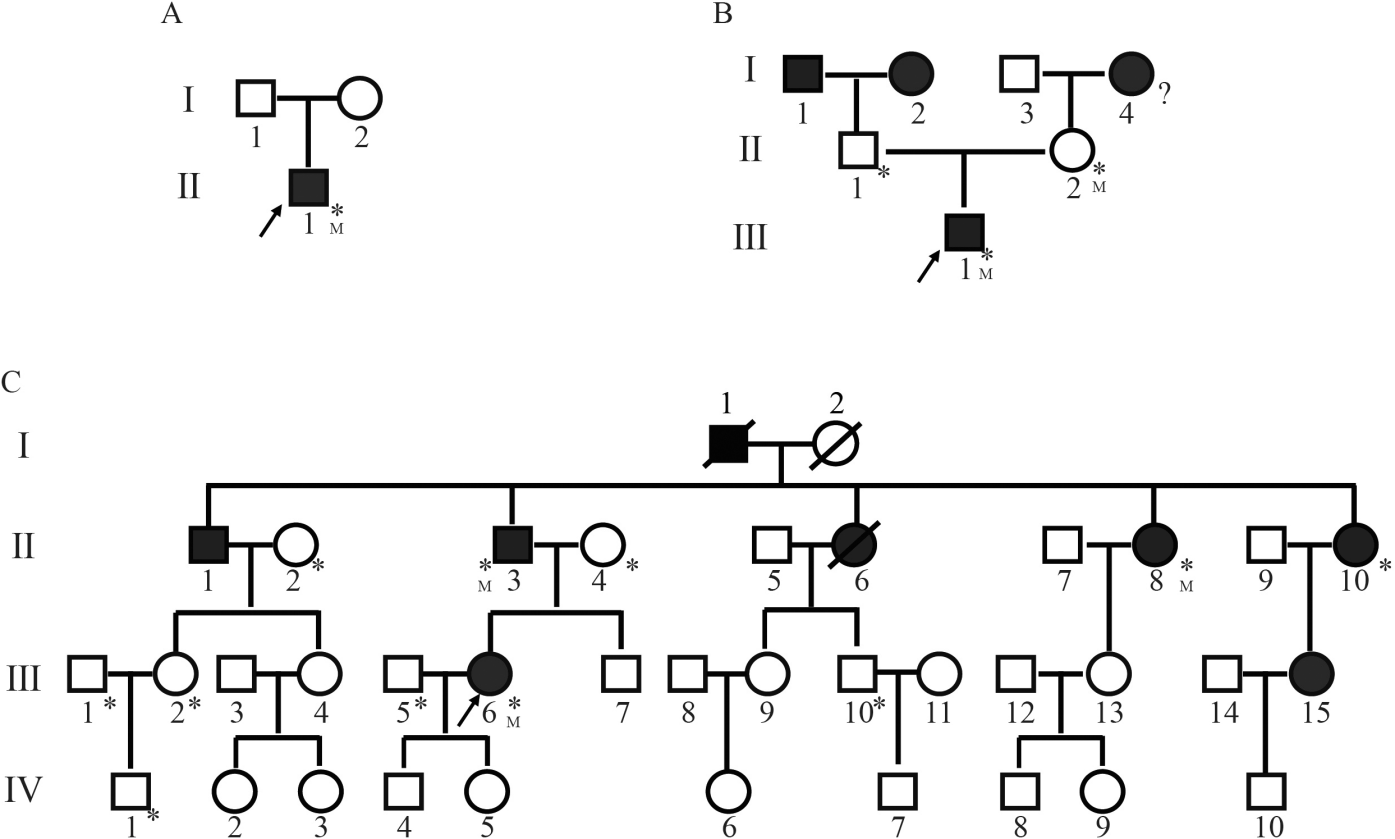
Family pedigrees of Probands A, B, and C with diabetes A) Pedigree of Proband A with diabetes. (B) Pedigree of Proband B with diabetes. (C) Pedigree of Proband C with diabetes. The proband is indicated by a black arrow. Black symbols denote individuals diagnosed with diabetes, while blank symbols represent unaffected individuals. Numbers marked with “M” indicate individuals harboring WFS1 gene mutations. Asterisks denote individuals with available blood samples.

Proband A, an adolescent male, presented with the complaint of “short penis for 2 weeks.” Additional self‐reported symptoms included slow growth and occasional fecal incontinence, along with binocular vision loss for the past 5 years. His medical history included type 1 diabetes mellitus (T1DM) for 3 years, bilateral sensorineural deafness for 2 years, and a body mass index (BMI) of 28.28 kg/m^2^. Laboratory tests revealed fasting blood glucose levels of 9.25 mmol/L, fasting insulin of 0.69 μU/mL, and glycated hemoglobin (HbA1c) of 8.60%. Urinalysis showed urinary glucose (++) and urinary ketone bodies (+). A breast ultrasound indicated male breast development (Table 1). Symptomatic treatments, including blood glucose control and nutritional support, were administered during hospitalization. Neither the father (I1) nor the mother (I2) had diabetes (Figure 1A, Table 1).

**TABLE 1:** Clinical characteristics and partial auxiliary examination results of Proband A, B, and C.

		Proband A	Proband B	Proband C	Reference range
Age of onset		15 years old	12 years old	35 years old	—
Clinical features	Diuresis	(+)	(+)	(−)	—
	Visual impairment	(+)	(−)	(−)	—
	Hearing impairment	(+)	(−)	(−)	—
	Fecal incontinence	(+)	(−)	(−)	—
	Short penis	(+)	(−)	(−)	—
	Obesity	(+)	(−)	(−)	—
Auxiliary inspection	Fasting blood glucose(mmol/L)	9.25	15	10.47	4.4–6.1
	Glycosylated hemoglobin (%)	8.6	11.1	10.5	4.0–6.0
	Insulin (μU/mL)	0.69	18.3	11	2.6–24.9
	C peptide (ng/mL)	0.12	2.81	2.8	1.1–4.4
	Neuron‐specific enolase (ng/mL)	19.1	—	—	0–16.3
	Urinary glucose (mg/dL)	250	< 160	< 160	< 160
	Urine output (mL/d)	—	2050	—	1000–2000
	Insulin auto‐antibodies	(−)	(−)	—	—

*Note:* (+), positive. (−), negative.

Proband B, also an adolescent, sought medical care due to “sticky urine for 1 year.” He had no symptoms of polyuria, dry mouth, polydipsia, weight loss, blindness, hearing impairment, or neurological symptoms such as numbness or pain in the extremities. Laboratory findings included fasting blood glucose of 15.00 mmol/L, HbA1c of 11.10%, and a daily urine volume of 2050 mL (Table 1). During hospitalization, treatment focused on blood glucose management. A family history revealed that the grandfather (I1), grandmother (I2), and great‐grandmother (I4) had diabetes, but both the father (II1) and mother (II2) did not (Figure 1B).

While both Proband A and Proband B were diagnosed with diabetes mellitus, Proband A showed additional clinical features, including visual impairment, hearing impairment, and delayed gonadal development. These complications made Proband A's overall condition more severe and the diagnostic and therapeutic process more challenging.

Proband C, an adult pregnant woman, presented to the Endocrine and Metabolic Disease Hospital affiliated with Shandong First Medical University with elevated blood glucose levels. During her last hospitalization, fasting blood glucose was measured at 10.47 mmol/L, HbA1c at 10.5%, and fasting insulin at 11 μU/L (Table 1). Proband C had previously been diagnosed with “gestational diabetes” on two occasions. During her second pregnancy, she developed diabetic ketoacidosis. Proband C's family history revealed seven additional members with diabetes, including the grandfather (I1), father (II3), uncle (II1), three aunts (II6, II8, II10), and cousin (III15) (Table 2, Figure 1C). None of these family members had a history of ketoacidosis, and three were treated with insulin. BMI values among the family members ranged from 19 to 27.7 kg/m^2^. Detailed clinical evaluations, including audiograms, annual ophthalmoscopy, and examinations for diabetes insipidus, did not reveal any features consistent with WS, such as hearing impairment, optic atrophy, or vision impairment (Table 2).

### 3.2 Genetic Test Results

**FIGURE 2:**
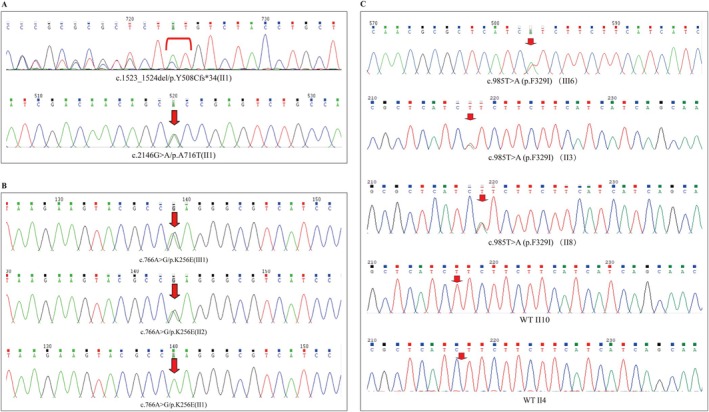
WFS1 gene sequencing results of family members (A–C) Sequencing results of the WFS1 gene for Proband A, Proband B, the parents of Proband B, Proband C, and family members of Proband C. The panels illustrate the mutation‐carrying status of WFS1 gene variants across different families.

Peripheral blood samples were collected from the probands for genetic testing, and the following *WFS1* mutations were identified. In Proband A, two *WFS1* mutations were detected: c.1523‐1524del/p.Y508Cfs*34 and c.2146G>A/p.A716T, both located in exon 7 (Figure 2A). The former, a frameshift mutation, results from the deletion of bases 1523 to 1524 in the coding region, causing a premature stop codon and truncation of the protein. The latter, a missense mutation, causes an amino acid substitution at position 716, replacing alanine with threonine (Figure 2A). Since no peripheral blood samples were obtained from the parents of Proband A, the inheritance pattern of these mutations could not be determined.

A heterozygous *WFS1* mutation (c.766A>G/p.K256E) was identified in Proband B. This missense mutation, located in exon 6, causes a substitution of lysine with glutamic acid at position 256. Family testing revealed that Proband B's mother (II2) also carried the heterozygous *WFS1* mutation c.766A>G/p.K256E, whereas his father (II1) was wild‐type (Figure 2B).

Proband C carried a heterozygous *WFS1* mutation c.985T>A/p.F329I. This missense mutation in exon 5 results in the substitution of phenylalanine with isoleucine at position 329. WES showed no further deletions or duplications in Proband C, apart from the *WFS1* mutation. Family testing in the three generations of Proband C's family revealed that II3 and II8 carried the mutation and had diabetes (Figures 1C, 2C). The mother (II4) was wild‐type and did not have diabetes. Interestingly, another aunt (II10) had diabetes but did not carry the genetic mutation (Figures 1C, 2C).

### 3.3 Pathogenicity Analysis and Confirmation of Mutant Genes

**FIGURE 3:**
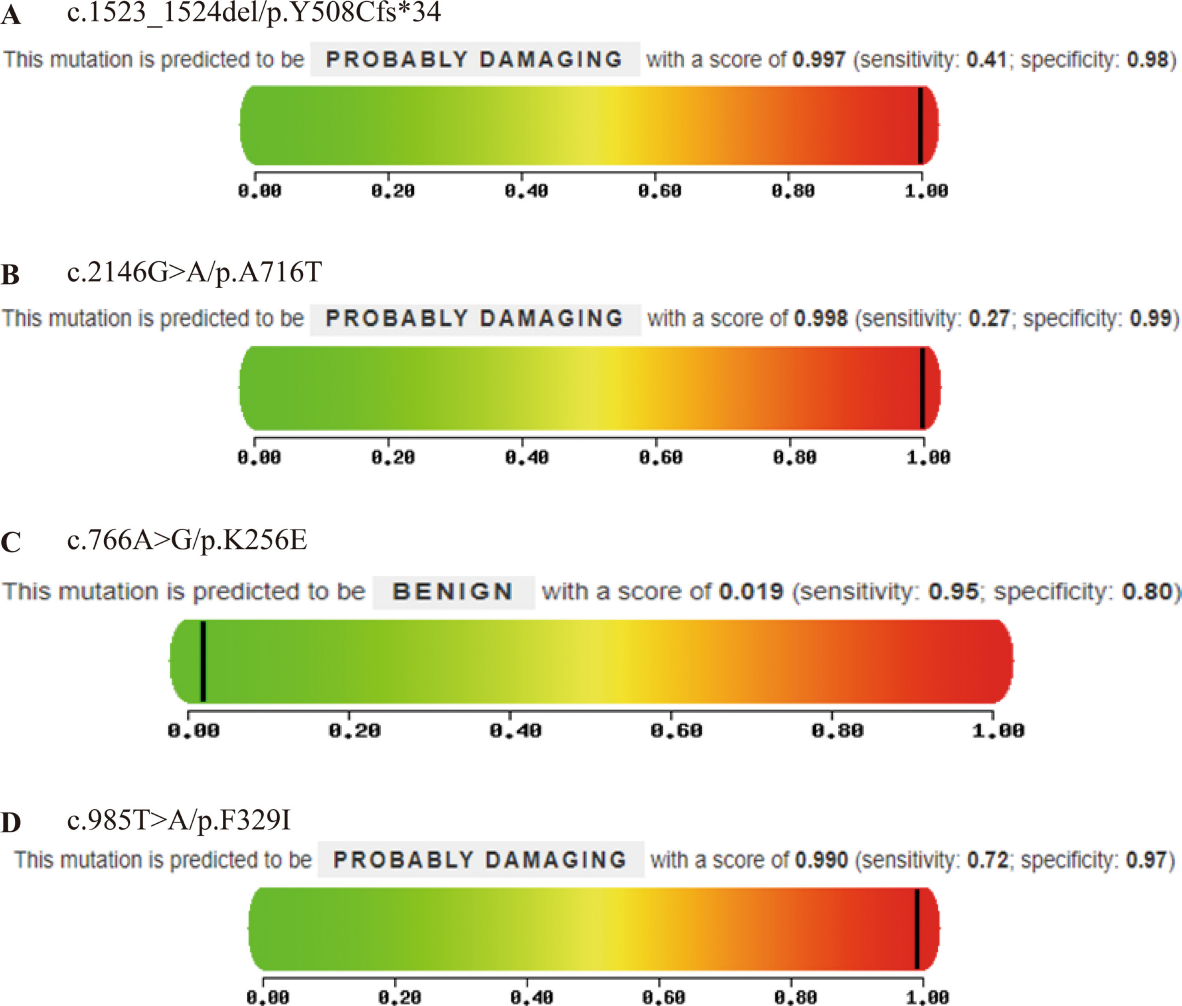
Pathogenicity prediction of WFS1 gene mutations using PolyPhen‐2 (A–B) PolyPhen‐2 prediction results for the mutations identified in Proband A. (C) PolyPhen‐2 prediction results for the mutations identified in Proband B. (D) PolyPhen‐2 prediction results for the mutations identified in Proband C.

Bioinformatics tools Mutation Taster and PolyPhen‐2 were used to predict the pathogenicity of the identified mutations. The results showed that in Proband A, the frameshift mutation (c.1523‐1524del/p.Y508Cfs*34) and the missense mutation (c.2146G>A/p.A716T) were both predicted to be pathogenic (Figure 3A, Table 3). In Proband B, the missense mutation (c.766A>G/p.K256E) was classified as pathogenic by Mutation Taster but benign by PolyPhen‐2 (Figure 3B, Table 3). In Proband C, the missense mutation (c.985T>A/p.F329I) was predicted to be pathogenic by both tools (Figure 3C, Table 3).

**TABLE 2:** Clinical data of Proband C's family members.

ID	Duration of diabetes (y)	BMI (kg/m^2^)	FC‐P (ng/mL)	HbA1C (%)	CRP (mg/L)	ALT (U/L)	AST (U/L)	LDH (U/L)	TG (mmol/L)	CH (mmol/L)	LDL‐CH (mmol/l)	HDL‐CH (mmol/L)	UA (μmol/L)	Cr (μmol/L)
II2	—	27.7	2.35	5.4	1.2	28	22	190	1.08	4.25	2.69	1.26	257	53.6
II3	20	19	9.62	7	0.5	13	11	181	1.3	4.65	2.91	1.1	342	846.2
II4	—	23	1.89	6.1	2.5	14	21	252	1.47	5.2	3.5	1.17	321	75
II8	1	23.1	2.02	6.6	0.5	74	36	244	1.31	4.7	2.73	1.52	257	43.8
II10	10	22	2.22	6.8	1.3	15	15	162	1.37	5.42	3.62	1.26	249	45.1
III1	—	23	1.94	5.1	0.3	19	16	220	2.88	4.32	2.24	0.96	381	72.6
III2	—	20	1.47	5.3	0.4	11	16	189	0.53	5.07	2.72	1.99	246	52.7
III5	—	31.1	2.62	5.8	3.7	52	30	227	1	5.6	4.27	1.02	325	63.1
**III6**	**5**	**24.2**	**2.8**	**10.5**	**3.05**	**49**	**26.2**	—	**1.2**	**5.35**	**3.4**	**1.38**	**334.7**	**59.7**
III10	—	38.4	3.46	5.7	2.6	59	27	214	2.54	6.03	4.37	1.16	498	80.5
IV1	—	17.5	2.45	5.5	0.6	11	14	222	1.45	4.9	3.36	1.18	474	65.7
Reference range	—	18.5–23.9	1.1–4.4	4–6	0–3	7–40	13–35	109–245	0.34–1.71	3.1–5.7	2.07–3.10	0.9–1.55	150–420	44–133

Abbreviations: ALT, alanine transaminase; AST, aspartate aminotransferase; BMI, body mass index; CH, cholesterol; Cr, creatinine; CRP, C‐reactive protein; FC‐P, fasting C‐peptide; HbA1C, glycated hemoglobin; HDL‐CH, high‐density lipoprotein cholesterol; LDH, lactate dehydrogenase; LDL‐CH, low‐density lipoprotein cholesterol; TG, triglyceride; UA, uric acid. *Note:* Bold indicates clinical data of Proband C.

**FIGURE 4:**
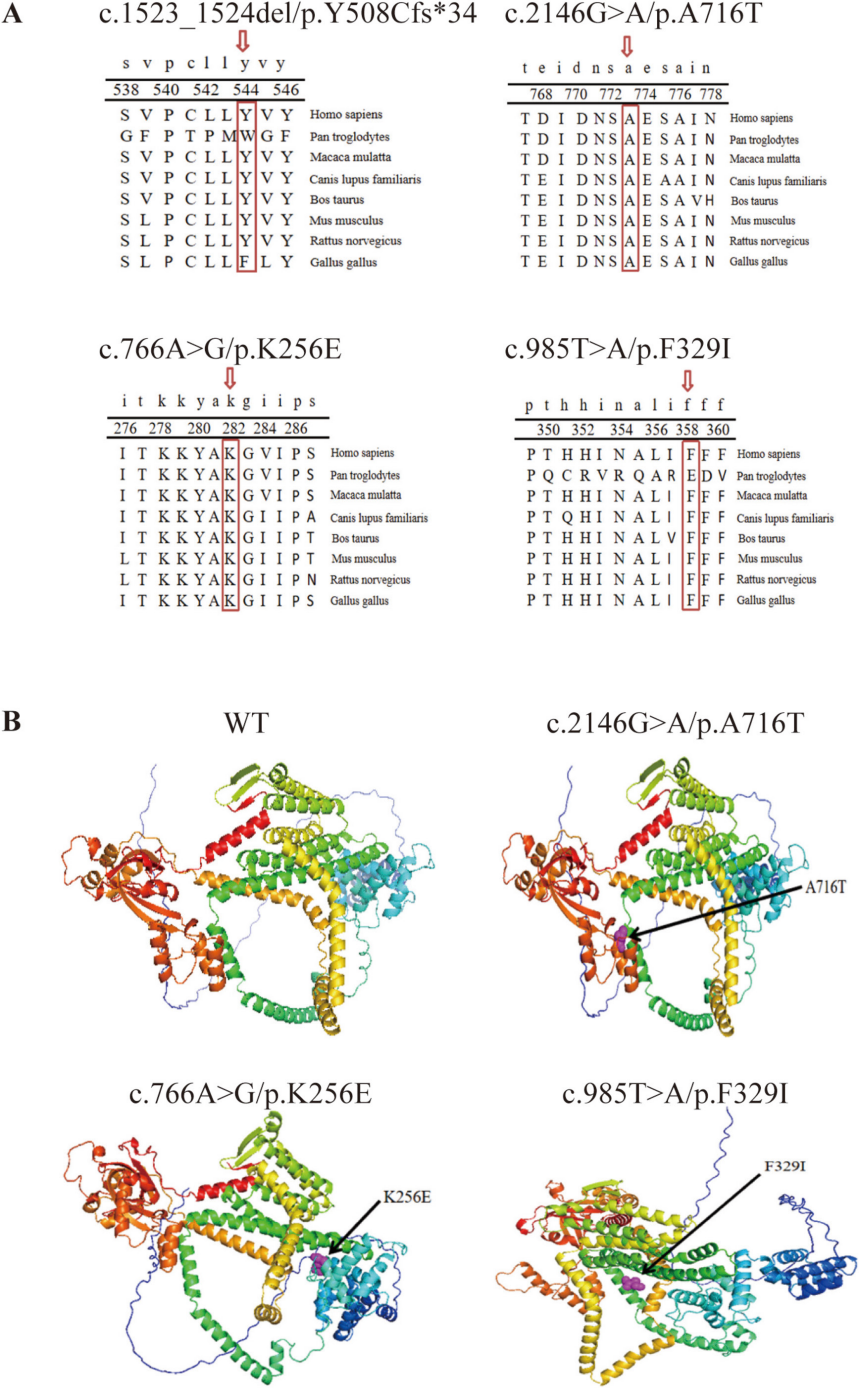
Multiple sequence alignment and three‐dimensional structure of Wolframin protein (A) Multiple sequence alignment of Wolframin protein across different species, highlighting conserved regions. Conserved sites are marked with red arrows. (B) Predicted structures of wild‐type and mutant Wolframin protein. The mutation site in the WFS1 protein is indicated by a purple sphere.

Conservation analysis using UGENE software indicated that all identified mutations (c.1523‐1524del/p.Y508Cfs*34, c.2146G>A/p.A716T, c.766A>G/p.K256E, and c.985T>A/p.F329I) showed high conservation across species, suggesting functional importance (Figure 4A). Homology modeling using the Swiss Model and PyMOL software visualized the spatial structure of the wolframin protein and the altered residues. The mutated residues were located within critical structural domains, likely interfering with protein function and molecular interactions (Figure 4B).

### 3.4 Summary of *WFS1* Genotype–Phenotype Relationship

**FIGURE 5:**
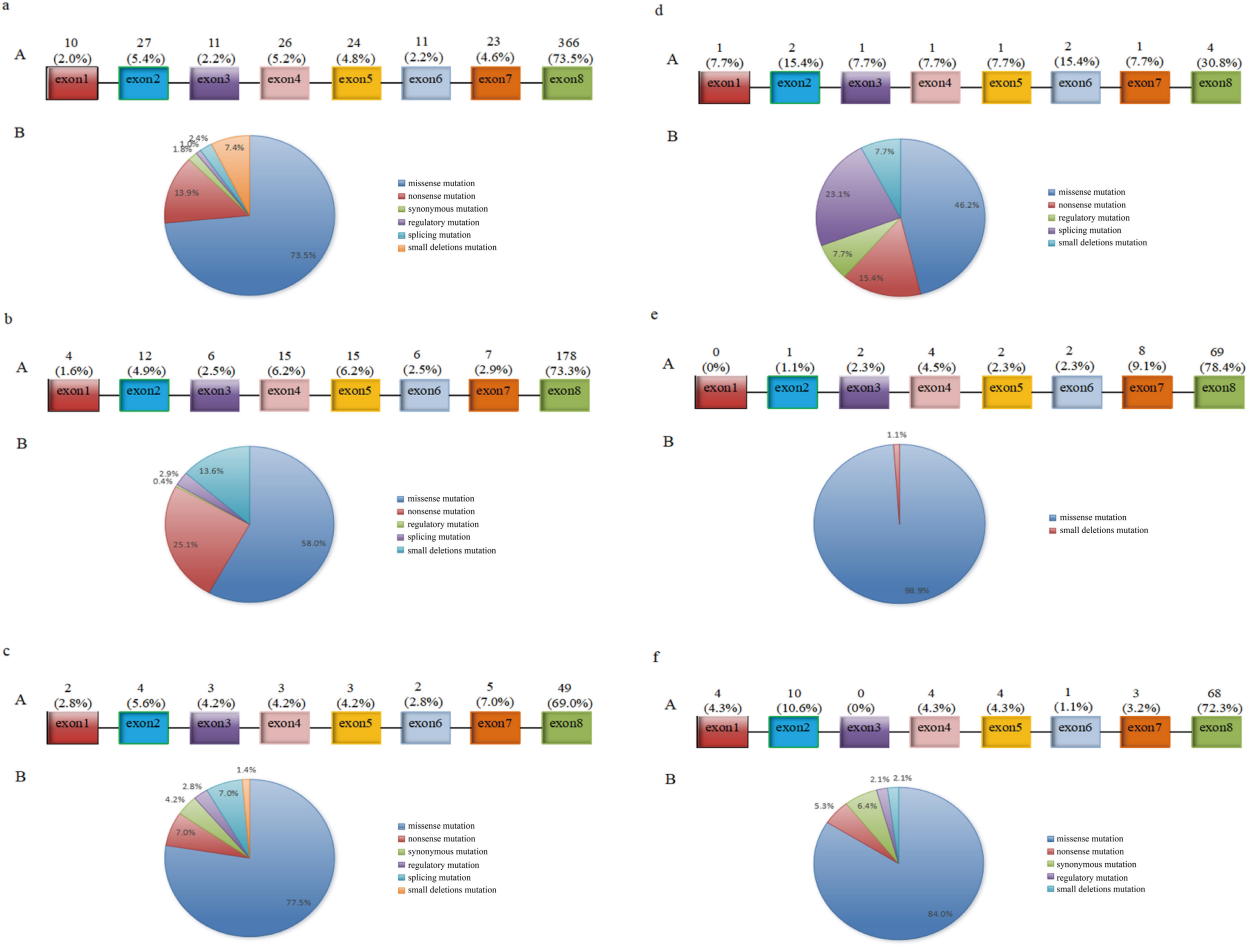
Distribution of WFS1 mutations across phenotypes and mutation types. (A) Overall distribution of WFS1 mutations. (B–F) Distribution of WFS1 mutations associated with specific phenotypes, including Wolfram syndrome (WS), diabetes, MODY, isolated hearing impairment, and other conditions (e.g., mental disorders, ocular abnormalities, ataxia, and developmental disorders). (a) Proportions of WFS1 mutations located in different exons. (b) Proportions of WFS1 mutations by mutation type.

A total of 498 reported *WFS1* mutations were analyzed, of which 366 (73.5%) were located in exon 8. Missense mutations constituted 73.5% of all mutation types. The associated clinical phenotypes included WS, type 1 diabetes, type 2 diabetes, MODY, hearing impairment, mental disorders, eye abnormalities, ataxia, and more. Among these, 243 mutations (48.8%) were associated with WS. Only 71 mutations (14.3%) were associated with diabetes, and of these, just 13 (2.6%) were linked specifically to MODY. Mutations manifesting solely as hearing impairment accounted for 17.7%, while those associated with mental disorders represented 5.8% (Figure 5).

## 4 Discussion

*WFS1‐*associated disorders encompass a spectrum of rare diseases that include WS, WFLS, and MODY. In this study, three families with *WFS1* gene mutations were analyzed, assessing the pathogenicity of the mutations and the clinical manifestations observed in the three probands. Among these, Family 3 represents the family with the largest number of reported affected members to date. The clinical phenotypes caused by *WFS1* gene mutations demonstrated heterogeneity across the three families, including manifestations of WS, WFLS, and MODY, as one of these families was identified as having MODY due to a *WFS1* mutation. Moreover, the genotype–phenotype relationships of all reported *WFS1* mutations were summarized. One novel mutation, c.766A>G/p.K256E, was discovered among the four identified mutations. The family of Proband C showed an apparent autosomal dominant inheritance pattern of adult diabetes, with WES identifying the c.985T>A/p.F329I mutation in the *WFS1* gene. Furthermore, a review of the literature revealed that inactivating mutations result in more severe WS phenotypes compared to missense mutations. This finding aligns with the observed phenotypic differences between Probands A and B in this study.

**TABLE 3:** Pathogenicity prediction of mutation sites.

WFS1 gene mutation	Mutation taster		Polygon‐2	
	Fraction	Result	Fraction	Result
c.1523_1524del/p.Y508Cfs*34	1	A	0.997	D
c.2146G>A/p.A716T	0.999	D	0.998	D
c.766A>G/p.K256E	0.949	D	0.019	B
c.985T>A/p.F329I	0.999	D	0.99	D

*Note:* Mutation Taster Prediction Results: A: Disease causing automatic; D: Disease causing; N: Polymorphism; P: Polymorphism automatic. Polygon‐2 forecast results: D: Probably damaging (≥ 0.957, probably damaging); P: Possibly damaging (0.453 ~ 0.956, possibly damaging); B: Benign (≤ 0.452, harmless).

The *WFS1* gene comprises eight exons, with exon 1 being non‐coding and exons 2–7 encoding smaller segments of the protein. Exon 8 is the largest, encoding the transmembrane and C‐terminal domains of the protein. According to the HGMD, 604 *WFS1* mutations have been reported. However, our analysis identified 498 mutations, including 365 missense mutations, 70 nonsense mutations, 9 synonymous mutations, 12 splicing mutations, 5 regulatory mutations, and 37 minor deletion mutations. The mutations analyzed were mainly distributed in exon 8, which is responsible for encoding critical functional domains of the protein [30]. Mutations in exon 1 predominantly consisted of regulatory mutations, whereas those in other exons were primarily missense mutations. The clinical phenotypes associated with *WFS1* mutations were diverse, with WS and diabetes (including common types of diabetes and MODY) being the most prevalent. Additional phenotypes included hearing impairment, mental health disorders (e.g., suicide, depression, schizophrenia, emotional disorders, and autism), eye abnormalities (e.g., cataracts, retinopathy, and iris defects), ataxia, and developmental disorders. Mutations in exon 8 resulted in the most complex and diverse clinical phenotypes, while mutations in exons 3 and 6 were associated with relatively fewer phenotypic presentations. In this study, the identified mutations were located in different exons: c.1523_1524del/p.Y508Cfs*34 and c.2146G>A/p.A716T in exon 7; c.766A>G/p.K256E in exon 6; and c.985T>A/p.F329I in exon 5.

The first mutation, c.1523_1524del/p.Y508Cfs*34, identified in Proband A, was predicted to be a pathogenic frameshift mutation through bioinformatics analysis. This mutation alters the amino acid sequence and causes premature termination of wolframin protein synthesis, potentially affecting its structure and function. However, the specific functional impacts of this mutation remain unclear. Another mutation, c.2146G>A/p.A716T, identified in the same proband, is a missense mutation previously reported as heterozygous in several families with sensorineural hearing loss [31, 32]. This mutation is associated with autosomal dominant inherited hearing loss [33]. Proband A had a complex clinical phenotype, including diabetes, bilateral optic atrophy, bilateral sensorineural deafness, diabetes insipidus, and fecal incontinence. The clinical phenotypes and genotype of patient A are helpful to the clinician in the diagnosis of WS. But we did not get the inheritance pattern of patient A's genes, because of no peripheral blood samples were obtained from the parents of Proband A, Proband A's overall condition is more severe. *WFS1* is identified as the causative gene for WS; the disease is progressive and is adversely affected by poor blood glucose control. Identifying the molecular genetic etiology provides early diagnostic confirmation. And this allows the subtle symptoms to be recognized and managed more appropriately.

In Proband B, the missense mutation c.766A>G/p.K256E was detected in the *WFS1* gene. This mutation was also found in his mother but was absent in his father. Unlike Proband A, Proband B had diabetes as the sole clinical manifestation, without visual or hearing impairments or other neurological symptoms. Besides WS, the *WFS1* gene is also implicated in WFLS, caused by heterozygous mutations in *WFS1*. Clinically, WFLS is characterized by the triad of optic atrophy (OA), diabetes mellitus (DM), and hearing impairment (HI), similarly to autosomal recessive WS, but there were no clear phenotype–genotype correlations [1, 34]. WFLS is distinguished from WS by its relatively milder phenotype, and patients with WFLS appear to have a different age at onset per symptom [1]. In the case of patient B, we inferred that he may be a WFLS, mainly because he had diabetes as the sole clinical manifestation, without visual or hearing impairments or other neurological symptoms. But as the disease develops, more information will be needed to separate WFLS from WS. In the future, blood samples must be taken from the grandparents for further testing to determine genetic phenotype.

Proband C carried the missense mutation c.985T>A/p.F329I, which may lead to changes in the amino acid sequence of wolframin. WES did not identify other relevant causative genes. In addition to diabetes, Proband C presented with dyslipidemia and menstrual irregularities. Three family members were identified as carriers of the c.985T>A/p.F329I mutation, but none had visual or auditory impairments or other neurological symptoms. While Proband C's uncle (II1) refused genetic testing, he also had diabetes. The clinical features of this family were consistent with the MODY genetic profile [35], supporting the conclusion that the *WFS1* c.985T>A/p.F329I mutation may act as a pathogenic factor for diabetes in this family, following an autosomal dominant inheritance pattern. MODY is a group of monogenic disorders characterized by an autosomal dominant, non‐insulin‐dependent form of diabetes that classically presents in adolescents or young adults [24]. In addition to clinical features, direct sequencing can diagnose MODY with up to 100% sensitivity. Proband C, with diabetes, has an obvious family history of diabetes, C peptide in the normal level. Although she was diagnosed with diabetes at the age of 35, she had no hearing loss, optic atrophy or visual impairment, and WES showed only the WFS1 mutation c.985T>A/p.F329I. And the *WFS1* variant (c.985T>A/p.F329I) segregated with diabetes and non‐diabetes individuals tested. We diagnosed patient C with MODY by combining the clinical phenotype and genotype of patient C.

This study highlights that different *WFS1* mutations are associated with different clinical manifestations and diagnoses. For instance, the complex clinical presentation of Proband A is consistent with the severe phenotypes caused by frameshift mutations. Previous studies have demonstrated that inactivating mutations, such as deletions, insertions, nonsense mutations, and splice site mutations, lead to more severe WS phenotypes compared to missense mutations [36]. Frameshift mutations, in particular, result in early termination of protein synthesis or the loss of large protein fragments, significantly impairing protein function and leading to severe diseases or even death [37]. The clinical differences observed between Probands A and B may be attributable to the distinct mutation types, with Proband A's frameshift mutation contributing to a more severe phenotype. Moreover, WS is a progressive disorder, and its full clinical spectrum may take time to manifest. This progression highlights the need for long‐term follow‐up to monitor for the emergence of further symptoms in patients with *WFS1* mutations.

Diabetes mellitus is a common clinical manifestation of disease caused by mutations in the *WFS1* gene and requires further differentiation on the basis of laboratory findings. In most cases, diabetes is the first presenting symptom of WS, with other systemic symptoms absent at the initial diagnosis. Due to its early onset, negative autoantibody status, and insulin dependency, WS‐related diabetes is frequently misdiagnosed as autoantibody‐negative T1DM. Subsequent manifestations, such as optic atrophy, may also be misinterpreted as complications of T1DM. As a result, the incidence of WS in children and adolescents is likely underestimated, leading to delays in accurate diagnosis and appropriate treatment [38, 39, 40, 41]. Several studies have highlighted distinct differences between WS and T1DM. Compared to T1DM patients, WS patients show lower daily insulin requirements, lower average HbA1c levels, lower incidence of diabetic ketoacidosis and microvascular complications, and higher incidence of severe hypoglycemia [42, 43]. Research by Zmyslowska et al. [44] further demonstrated that WS patients retain endogenous insulin secretion, which reduces blood glucose variability compared to T1DM patients. This retained insulin secretion slows the progression to complete insulin deficiency, allowing for better glycemic control in WS patients. However, WS patients experience severe deficits in pancreatic β cell count or function earlier in life [45], resulting in the rapid progression of WS‐related diabetes. To reduce the misdiagnosis rate and ensure timely and appropriate treatment for WS patients, routine ophthalmic screening and *WFS1* gene testing are recommended in suspected WS cases, particularly in T1DM patients who retain endogenous insulin secretion [38, 39, 40, 41]. These diagnostic measures will improve the accuracy of WS identification and facilitate early intervention. Maturity‐onset diabetes of the young (MODY) is a type of monogenic diabetes. Although many subtypes of MODY are recognized, misdiagnosis of MODY as type 1 or type 2 diabetes is common due to overlap of clinical characteristics, high cost, and limitations of genetic testing.

However, structural and functional changes in the wolframin protein, caused by *WFS1* mutations, primarily contribute to diabetes by impairing proinsulin processing and insulin secretion, inducing ER‐mediated pancreatic β‐cell death, and causing mitochondrial dysfunction [28]. The clinical manifestations of WS share similarities with mitochondria‐related diseases, leading to its initial characterization as a mitochondrial disorder [46]. However, this hypothesis has been questioned since the wolframin protein is localized to the ER [6, 7]. Angebault et al. [47] demonstrated that wolframin forms a complex with neuronal calcium sensor 1 and inositol 1,4,5‐triphosphate receptors, facilitating calcium ion transfer between the ER and mitochondria. Defects in wolframin reduce mitochondrial calcium uptake, destabilize MAM, and impair autophagy and mitophagy [30, 34]. Neuronal mitochondria play a critical role in glucose metabolism, and structural or functional defects in wolframin may disrupt glucose homeostasis via neuronal mitochondrial dysfunction, contributing to the onset and progression of diabetes [48, 49].

In conclusion, diabetes is the most common symptom of *WFS1‐*associated disorders, often indistinguishable from other forms of diabetes based on clinical symptoms and routine examinations. *WFS1*‐related diabetes typically has an early onset, with ~95% of cases manifesting before the age of 25, primarily affecting children and adolescents [25]. Genetic testing should be conducted promptly for suspected WS cases to identify the pathogenic mutations, establish a definitive diagnosis, and initiate early intervention. Early genetic counseling can also help prevent the inheritance of related conditions in future generations through eugenic and childcare strategies [18]. Recent studies [50, 51, 52] have suggested that glucagon‐like peptide‐1 receptor agonists, rapamycin, pioglitazone, and dantrolene, can mitigate pancreatic stress by reducing ER stress‐induced β‐cell death, thereby slowing disease progression. The *WFS1* mutant disrupts the receptor splice site and affects the pattern of mRNA splicing, which leads to β‐cell apoptosis and promotes β‐cell dedifferentiation and loss of normal insulin secretion, leading to diabetes [28, 45]. In the nervous system, *WFS1* mutations can cause neurodegeneration, optic nerve energetic failure and neuroinflammation, and oligodendrocyte ER stress [53, 54, 55]. Given the complexity of the pathogenesis of WFS1 mutations, early genetic testing is important and may also aid in the search and development of effective treatments [28, 29, 30].

This study presented three families with *WFS1* mutations, highlighting significant differences in mutation types and clinical manifestations. Patients with frameshift mutations had more severe and complex clinical features, consistent with previous literature. Moreover, the largest *WFS1*‐associated diabetes pedigree to date was reported, showing a genetic pattern consistent with MODY. The genotype–phenotype relationship of *WFS1* mutations remains complex and incompletely understood.

The limitation of this study is the lack of functional validation of the *WFS1* mutations. Further research is needed to elucidate the structural and functional roles of the *WFS1* gene and wolframin protein. Investigating the genetic, pathophysiological, and clinical characteristics of WS, WFLS, and MODY will improve diagnostic accuracy, inform targeted treatment strategies, and improve patient quality of life. The understanding and management of *WFS1*‐related disorders can be advanced by addressing these challenges.

About the spectrum of inherited diseases associated with *WFS1*, there are still many unanswered questions. What is the exact inheritance pattern? Why do different *WFS1* mutations cause different clinical presentations? Why does the disease vary from person to person? The different roles of wolframine at different developmental stages or tissue‐ and organ‐specific pathophysiological processes may provide answers to these questions. To identify the exact pathophysiological mechanisms within the different organs affected in *WFS1*‐associated diabetes, further functional and clinical studies are needed.

## 5 Conclusion

Summary, our study expands the mutation spectrum of the *WFS1* gene with one novel mutation, confirming that inactivating mutations and benign missense mutations are associated with more severe WS phenotypes compared to purely pathogenic missense mutations. Moreover, c.985T>A/p.F329I has been validated as a mutation associated with MODY. Finally, summarizing the *WFS1* genotype–phenotype relationship, the clinical presentation associated with mutations in the *WFS1* gene varies between patients, possibly due to differences in the mutation sites.

## Author Contributions

All authors were involved in the conduct of the study, in interpreting the results, and in revising and correcting the manuscript. Dong Wang, YiPing Cheng, Ping Shi, ChunXiao Yu, XiaoHong Li individually collected the clinical data of the patients. Hui Zhao, Wei Hou collected the samples for sequencing detection. ZhenKui Guo, Chao Xu, QingBo Guan planned and conducted the research. ChangQing Liu, HangYu Fang analysis the data and wrote the manuscript. All authors read and approved the final version of the manuscript.

## Conflicts of Interest

The authors declare no conflicts of interest.
